# Crystal structure of the tripeptide *N*-(benzyl­oxycarbon­yl)glycylglycyl-l-norvaline

**DOI:** 10.1107/S205698901500393X

**Published:** 2015-02-28

**Authors:** Sumesh Nicholas

**Affiliations:** a Dept. of Physics, Indian Institute of Science, Bangalore 560012, India

**Keywords:** crystal structure, peptide, conformation, norvaline, glycine, hydrogen bonding

## Abstract

The title tripeptide, C_17_H_23_N_3_O_6_, contains a nonproteinogenic C-terminal amino acid residue, norvaline, which is an isomer of the amino acid valine. Norvaline, unlike valine, has an unbranched side chain. The mol­ecule has a Gly–Gly segment which adopts an extended conformation. The norvaline residue also adopts an extended backbone conformation while its side chain has a *g* ^+^ *t* conformation. In the crystal lattice, N—H⋯O and O—H⋯O hydrogen bonds stabilize the packing. Mol­ecules translated along the crystallographic *a* axis associate through an N—H⋯O hydrogen bond. The remaining three hydrogen bonds are between mol­ecules related by a *2*_1_ screw axis.

## Related literature   

For information on the amino acid norvaline, see: Kisumi, Sugiura & Chibata (1976 ▸); Kisumi, Sugiura, Kato & Chibata (1976 ▸); Alvarez-Carreño *et al.* (2013 ▸). For the conformation of glycine residues in proteins and peptides, see: Ramakrishnan & Srinivasan (1990 ▸). For examples of the conformational flexibility of Gly–Gly segments in peptides, see: Smith *et al.* (1978 ▸); Karle *et al.* (1983 ▸); Aubry *et al.* (1989 ▸).
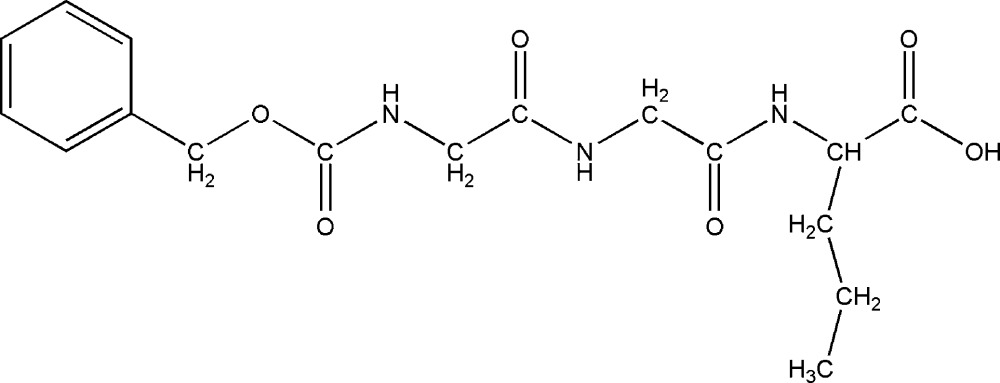


## Experimental   

### Crystal data  

C_17_H_23_N_3_O_6_
*M* *_r_* = 365.38
Orthorhombic, 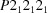

*a* = 4.9857 (6) Å
*b* = 19.372 (2) Å
*c* = 19.476 (2) Å
*V* = 1881.1 (4) Å^3^
*Z* = 4
Mo *K*α radiation
μ = 0.10 mm^−1^
*T* = 293 K
0.6 × 0.1 × 0.1 mm

### Data collection  

Bruker Kappa APEXII CCD diffractometer
Absorption correction: multi-scan (*SADABS*; Bruker, 2001 ▸) *T*_min_ = 0.635, *T*_max_ = 0.746
33216 measured reflections
2747 independent reflections
1421 reflections with *I* > 2σ(*I*)
*R*_int_ = 0.156

### Refinement  

*R*[*F* ^2^ > 2σ(*F* ^2^)] = 0.083
*wR*(*F* ^2^) = 0.249
*S* = 1.05
2747 reflections
243 parameters
5 restraints
H atoms treated by a mixture of independent and constrained refinement
Δρ_max_ = 0.32 e Å^−3^
Δρ_min_ = −0.23 e Å^−3^

### 

Data collection: *APEX2* (Bruker, 2007 ▸); cell refinement: *SAINT-Plus* (Bruker, 2007 ▸); data reduction: *SAINT-Plus*; program(s) used to solve structure: *SHELXS97* (Sheldrick, 2008 ▸); program(s) used to refine structure: *SHELXL97* (Sheldrick, 2008 ▸); molecular graphics: *ORTEP-3 for Windows* (Farrugia, 2012 ▸) and *Mercury* (Macrae *et al.*, 2006 ▸); software used to prepare material for publication: *SHELXL97*.

## Supplementary Material

Crystal structure: contains datablock(s) global, I. DOI: 10.1107/S205698901500393X/rz5147sup1.cif

Structure factors: contains datablock(s) I. DOI: 10.1107/S205698901500393X/rz5147Isup2.hkl

Supporting information file. DOI: 10.1107/S205698901500393X/rz5147Isup3.docx

Supporting information file. DOI: 10.1107/S205698901500393X/rz5147Isup4.cml

**Fig. 1.**
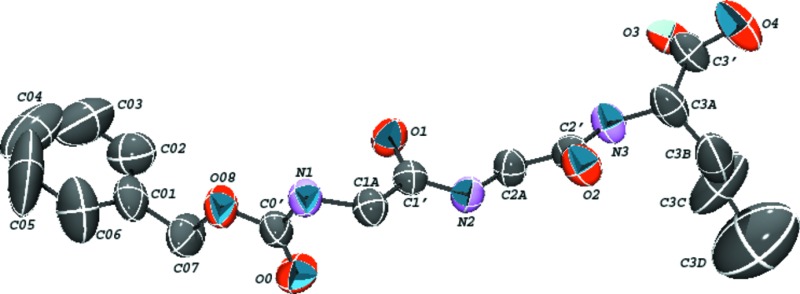
Thermal ellipsoid plot of the title compound drawn at the 50% probability level. Hydrogen atoms are omitted for clarity.

**Fig. 2.**
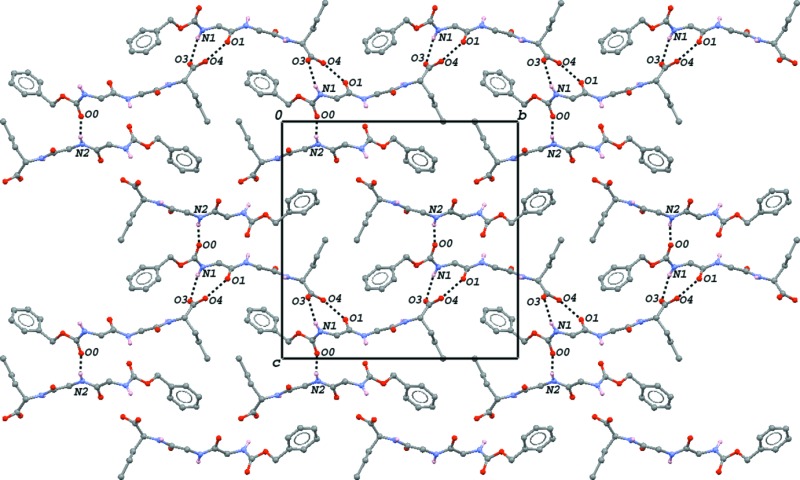
Crystal packing of the title compound viewed down the a axis. Hydrogen bonds are represented as dotted lines. Hydrogen atoms, except those involved in hydrogen bonds, are omitted for clarity.

. DOI: 10.1107/S205698901500393X/rz5147fig1.tif
Thermal ellipsoid plot of the title compound drawn at the 50% probability level. Hydrogen atoms are omitted for clarity.

a . DOI: 10.1107/S205698901500393X/rz5147fig2.tif
Crystal packing of the title compound viewed down the *a* axis. Hydrogen bonds are represented as dotted lines. Hydrogen atoms, except those involved in hydrogen bonds, are omitted for clarity.

CCDC reference: 1051240

Additional supporting information:  crystallographic information; 3D view; checkCIF report

## Figures and Tables

**Table 1:** Hydrogen-bond geometry (, )

*D*H*A*	*D*H	H*A*	*D* *A*	*D*H*A*
N1H1O3^i^	0.86	2.47	3.061(6)	127
N2H2O0^ii^	0.86	2.06	2.891(6)	164
N3H3O2^iii^	0.96(7)	2.36(7)	3.268(6)	159(5)
O4H4O1^iv^	0.82	1.83	2.593(5)	153

Symmetry codes: (i) 
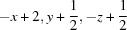
; (ii) 
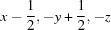
; (iii) 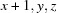; (iv) 
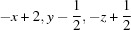
.

## supplementary crystallographic information

### S1. Chemical context

Norvaline is a non-proteinogenic amino­acid with an unbranched side chain. It is an isomer of the branched chain amino­acid valine. It is postulated that norvaline has been an abundant protein component during primitive stages of cell evolution (Alvarez-Carreño *et al.*, 2013). Norvaline is formed as a byproduct during isoleucine fermentation from threonine by *Serratia marcescens* (Kisumi, Sugiura & Chibata, 1976; Kisumi, Sugiura, Kato & Chibata, 1976). The title peptide contains a Gly-Gly segment. This structural study was undertaken as part of an endeavour to understand the conformational flexibility of consecutive glycine segments in short peptides. Due to the conformational freedom of glycine residues they are increasingly found in turns (Ramakrishnan & Srinivasan, 1990). In various polymorphic forms of Tyr-Gly-Gly-Phe-Leu, the Gly-Gly segment adopts extended conformation, type-I' β-turn and 3_10_ helical structures (Karle *et al.*, 1983; Smith & Griffin, 1978; Aubry *et al.*, 1989). This demonstrates the conformational flexibility of consecutive glycine sequences.

### S2. Structural commentary

The Gly-Gly segment of the protected tripeptide has an extended conformation with Gly(1) adopting torsion angle values *φ*_1_ = 76.2 (7)° and *ψ*_1_ = -166.6 (4)° and Gly(2) adopting torsion angle values *φ*_2_ = 133.1 (5)° and *ψ*_2_ = -175.5 (5)°. The norvaline residue adopts an extended conformation with torsion angle values *φ*_3_ = -152.6 (6)° and *ψ*_3_ = 165.6 (6)°. There are no intra­molecular hydrogen bonds which stabilize the backbone conformation of the peptide molecule. The side chain of norvaline adopts a *g*^+^*t* conformation.

### S3. Supra­molecular features

The packing in the crystal structure is stabilized by four inter­molecular hydrogen bonds (Table 1). Molecules translated along the crystallographic *a* axis associate through a N—H···O hydrogen bond. The remaining three hydrogen bonds are between molecules related by a *2*_1_ screw axis.

### S4. Synthesis and crystallization

The title compound was purchased commercially. Needle-shaped crystals of the title compound were obtained by slow evaporation from methanol/water (1:1 *v*/*v*) solution.

### S5. Refinement

The H-atoms bonded to N3 and C3A could be located from a difference Fourier map and refined freely. The remaining H-atoms were fixed geometrically in calculated positions and included in the refinement using a riding model approximation. The C—H distances were fixed at 0.97, 0.96 and 0.93 Å in case of hydrogens attached to methyl­ene, methyl and aromatic carbon atoms, respectively. N–H and O–H distances were fixed at 0.86 and 0.82 Å, respectively. The isotropic displacement parameters *U*_iso_ for hydrogen atoms were set at 1.5 times the *U*_eq_ of the carrier atoms in case of methyl groups and hydroxyl groups. In case of hydrogens attached to aromatic carbons, methyl­ene carbons and nitro­gen atoms, *U*_iso_ was set at 1.2 times the *U*_eq_ of the carrier atoms. The anisotropic displacement parameters of the carbon atoms C3A, C3B, C3C and C3D were restrained to be equal within a standard uncertainty of 0.01Å^2^ using the DELU command in *SHELXL97* (Sheldrick, 2008). In the absence of significant anomalous scattering effects, 1967 Friedel pairs were merged. The absolute configuration was known for the purchased material. The relatively high value of *R*int (0.12) is due to the poor quality of the crystal available.

### Crystal data

**Table:**

C_17_H_23_N_3_O_6_	*Z* = 4
*M**_r_* = 365.38	*F*(000) = 776
Orthorhombic, *P*2_1_2_1_2_1_	*D*_x_ = 1.290 Mg m^−^^3^
Hall symbol: P 2ac 2ab	Mo *K*α radiation, λ = 0.71073 Å
*a* = 4.9857 (6) Å	µ = 0.10 mm^−^^1^
*b* = 19.372 (2) Å	*T* = 293 K
*c* = 19.476 (2) Å	Needle-shaped, colourless
*V* = 1881.1 (4) Å^3^	0.6 × 0.1 × 0.1 mm

### Data collection

**Table:**

Bruker Kappa APEXII CCD diffractometer	2747 independent reflections
Radiation source: fine-focus sealed tube	1421 reflections with *I* > 2σ(*I*)
Graphite monochromator	*R*_int_ = 0.156
φ and ω scans	θ_max_ = 28.4°, θ_min_ = 1.5°
Absorption correction: multi-scan (*SADABS*; Bruker, 2001)	*h* = −5→6
*T*_min_ = 0.635, *T*_max_ = 0.746	*k* = −25→25
33216 measured reflections	*l* = −23→26

### Refinement

**Table:**

Refinement on *F*^2^	Primary atom site location: structure-invariant direct methods
Least-squares matrix: full	Secondary atom site location: difference Fourier map
*R*[*F*^2^ > 2σ(*F*^2^)] = 0.083	Hydrogen site location: inferred from neighbouring sites
*wR*(*F*^2^) = 0.249	H atoms treated by a mixture of independent and constrained refinement
*S* = 1.05	*w* = 1/[σ^2^(*F*_o_^2^) + (0.1213*P*)^2^ + 0.3601*P*] where *P* = (*F*_o_^2^ + 2*F*_c_^2^)/3
2747 reflections	(Δ/σ)_max_ < 0.001
243 parameters	Δρ_max_ = 0.32 e Å^−^^3^
5 restraints	Δρ_min_ = −0.23 e Å^−^^3^

### Special details

**Table:**

Geometry. All e.s.d.'s (except the e.s.d. in the dihedral angle between two l.s. planes) are estimated using the full covariance matrix. The cell e.s.d.'s are taken into account individually in the estimation of e.s.d.'s in distances, angles and torsion angles; correlations between e.s.d.'s in cell parameters are only used when they are defined by crystal symmetry. An approximate (isotropic) treatment of cell e.s.d.'s is used for estimating e.s.d.'s involving l.s. planes.
Refinement. Refinement of *F*^2^ against ALL reflections. The weighted *R*-factor *wR* and goodness of fit *S* are based on *F*^2^, conventional *R*-factors *R* are based on *F*, with *F* set to zero for negative *F*^2^. The threshold expression of *F*^2^ > σ(*F*^2^) is used only for calculating *R*-factors(gt) *etc*. and is not relevant to the choice of reflections for refinement. *R*-factors based on *F*^2^ are statistically about twice as large as those based on *F*, and *R*- factors based on ALL data will be even larger.

### Fractional atomic coordinates and isotropic or equivalent isotropic displacement parameters (Å^2^)

**Table:**

	*x*	*y*	*z*	*U*_iso_*/*U*_eq_	
C02	1.389 (2)	0.5256 (5)	0.1660 (5)	0.118 (3)	
H02	1.4846	0.4846	0.1698	0.141*	
C3B	0.793 (2)	−0.1324 (4)	0.1074 (5)	0.120 (3)	
H3B1	0.7123	−0.1758	0.1212	0.144*	
H3B2	0.6699	−0.1099	0.0759	0.144*	
C06	1.051 (3)	0.5922 (5)	0.1164 (6)	0.161 (4)	
H06	0.9095	0.5983	0.0860	0.194*	
C3C	1.050 (3)	−0.1472 (6)	0.0703 (7)	0.177 (5)	
H3C1	1.1610	−0.1754	0.1001	0.212*	
H3C2	1.1434	−0.1037	0.0638	0.212*	
C04	1.327 (5)	0.6385 (11)	0.2032 (8)	0.205 (11)	
H04	1.3823	0.6749	0.2308	0.246*	
C05	1.131 (5)	0.6467 (6)	0.1615 (11)	0.227 (10)	
H05	1.0399	0.6885	0.1605	0.272*	
C03	1.449 (4)	0.5791 (9)	0.2068 (6)	0.170 (6)	
H03	1.5833	0.5736	0.2394	0.204*	
C3D	1.033 (5)	−0.1837 (12)	−0.0004 (9)	0.326 (13)	
H3D1	1.2106	−0.1923	−0.0174	0.489*	
H3D2	0.9385	−0.1546	−0.0322	0.489*	
H3D3	0.9389	−0.2266	0.0045	0.489*	
H3	1.140 (14)	−0.013 (3)	0.156 (3)	0.082 (18)*	
H3A	0.634 (15)	−0.076 (3)	0.184 (3)	0.085 (18)*	
O3	1.2086 (8)	−0.1099 (2)	0.2416 (2)	0.0781 (11)	
O2	0.5636 (8)	0.0281 (2)	0.1214 (2)	0.0778 (11)	
N3	0.9505 (9)	−0.0208 (2)	0.1555 (3)	0.0688 (12)	
C2A	0.9611 (10)	0.0969 (2)	0.1159 (3)	0.0652 (13)	
H2A1	1.0607	0.1104	0.1565	0.078*	
H2A2	1.0892	0.0875	0.0796	0.078*	
N1	0.6659 (10)	0.3373 (2)	0.1138 (2)	0.0674 (11)	
H1	0.5879	0.3575	0.1477	0.081*	
C2'	0.8037 (10)	0.0320 (3)	0.1312 (3)	0.0593 (12)	
O1	0.9811 (8)	0.23128 (19)	0.1640 (2)	0.0730 (10)	
O08	0.9223 (10)	0.43006 (18)	0.1063 (2)	0.0864 (13)	
C1'	0.8092 (10)	0.2157 (2)	0.1204 (3)	0.0586 (12)	
O4	0.8419 (9)	−0.1744 (2)	0.2531 (3)	0.1091 (18)	
H4	0.9301	−0.1940	0.2828	0.164*	
O0	0.9319 (11)	0.3519 (2)	0.0213 (2)	0.0946 (15)	
C1A	0.5999 (11)	0.2674 (2)	0.0977 (3)	0.0645 (13)	
H1A1	0.5757	0.2635	0.0484	0.077*	
H1A2	0.4304	0.2560	0.1193	0.077*	
N2	0.7901 (9)	0.1528 (2)	0.0953 (2)	0.0652 (11)	
H2	0.6687	0.1447	0.0650	0.078*	
C3'	0.9838 (11)	−0.1230 (3)	0.2268 (3)	0.0727 (15)	
C01	1.1876 (17)	0.5311 (3)	0.1188 (4)	0.092 (2)	
C0'	0.8478 (13)	0.3711 (3)	0.0767 (3)	0.0683 (14)	
C3A	0.8252 (14)	−0.0865 (3)	0.1714 (4)	0.0841 (17)	
C07	1.1156 (18)	0.4727 (3)	0.0718 (4)	0.100 (2)	
H07A	1.0404	0.4905	0.0294	0.120*	
H07B	1.2742	0.4460	0.0607	0.120*	

### Atomic displacement parameters (Å^2^)

**Table:**

	*U*^11^	*U*^22^	*U*^33^	*U*^12^	*U*^13^	*U*^23^
C02	0.120 (6)	0.132 (7)	0.101 (6)	0.005 (6)	0.001 (6)	0.002 (5)
C3B	0.131 (6)	0.083 (4)	0.145 (6)	−0.026 (4)	−0.064 (5)	0.029 (4)
C06	0.163 (9)	0.094 (6)	0.228 (12)	0.020 (7)	0.003 (11)	0.006 (7)
C3C	0.150 (8)	0.165 (9)	0.215 (11)	0.058 (8)	−0.072 (7)	−0.103 (9)
C04	0.26 (2)	0.206 (18)	0.151 (12)	−0.117 (19)	0.104 (14)	−0.084 (13)
C05	0.26 (2)	0.080 (7)	0.34 (3)	−0.005 (11)	0.09 (2)	−0.068 (11)
C03	0.191 (13)	0.209 (14)	0.110 (8)	−0.065 (13)	0.013 (8)	−0.043 (9)
C3D	0.30 (2)	0.42 (3)	0.255 (19)	0.06 (3)	−0.050 (19)	−0.17 (2)
O3	0.062 (2)	0.103 (3)	0.069 (2)	0.005 (2)	0.0027 (19)	0.023 (2)
O2	0.062 (2)	0.078 (2)	0.093 (3)	0.0043 (19)	−0.012 (2)	0.018 (2)
N3	0.052 (2)	0.062 (3)	0.092 (3)	0.004 (2)	−0.009 (2)	0.017 (2)
C2A	0.059 (3)	0.057 (3)	0.080 (3)	0.004 (2)	−0.010 (3)	0.001 (2)
N1	0.079 (3)	0.056 (2)	0.067 (3)	0.003 (2)	0.019 (2)	−0.0038 (19)
C2'	0.052 (3)	0.065 (3)	0.061 (3)	0.008 (2)	−0.008 (2)	−0.004 (2)
O1	0.069 (2)	0.071 (2)	0.079 (2)	−0.003 (2)	−0.016 (2)	−0.0113 (18)
O08	0.112 (3)	0.059 (2)	0.088 (3)	−0.015 (2)	0.034 (3)	−0.0024 (18)
C1'	0.052 (2)	0.061 (3)	0.063 (3)	−0.004 (2)	0.012 (3)	0.001 (2)
O4	0.070 (2)	0.108 (3)	0.150 (5)	0.000 (3)	−0.012 (3)	0.067 (3)
O0	0.120 (4)	0.094 (3)	0.070 (3)	−0.017 (3)	0.036 (3)	−0.013 (2)
C1A	0.063 (3)	0.059 (3)	0.071 (3)	0.003 (3)	0.006 (3)	0.001 (2)
N2	0.066 (2)	0.058 (2)	0.072 (3)	0.006 (2)	−0.018 (2)	−0.0061 (19)
C3'	0.053 (3)	0.075 (3)	0.090 (4)	0.004 (3)	0.001 (3)	0.025 (3)
C01	0.111 (5)	0.060 (3)	0.105 (5)	−0.012 (4)	0.042 (5)	0.008 (3)
C0'	0.081 (3)	0.057 (3)	0.067 (3)	−0.005 (3)	0.011 (3)	0.005 (2)
C3A	0.067 (3)	0.074 (4)	0.111 (5)	−0.005 (3)	−0.021 (4)	0.032 (3)
C07	0.126 (6)	0.083 (4)	0.090 (4)	−0.039 (4)	0.032 (4)	−0.004 (3)

### Geometric parameters (Å, º)

**Table:**

C02—C03	1.341 (15)	N3—H3	0.96 (7)
C02—C01	1.364 (12)	C2A—N2	1.435 (6)
C02—H02	0.9300	C2A—C2'	1.511 (7)
C3B—C3C	1.501 (16)	C2A—H2A1	0.9700
C3B—C3A	1.540 (11)	C2A—H2A2	0.9700
C3B—H3B1	0.9700	N1—C0'	1.333 (7)
C3B—H3B2	0.9700	N1—C1A	1.427 (6)
C06—C01	1.365 (12)	N1—H1	0.8600
C06—C05	1.428 (19)	O1—C1'	1.244 (6)
C06—H06	0.9300	O08—C0'	1.331 (6)
C3C—C3D	1.551 (17)	O08—C07	1.436 (7)
C3C—H3C1	0.9700	C1'—N2	1.317 (6)
C3C—H3C2	0.9700	C1'—C1A	1.513 (7)
C04—C05	1.28 (3)	O4—C3'	1.324 (7)
C04—C03	1.30 (2)	O4—H4	0.8200
C04—H04	0.9300	O0—C0'	1.216 (6)
C05—H05	0.9300	C1A—H1A1	0.9700
C03—H03	0.9300	C1A—H1A2	0.9700
C3D—H3D1	0.9600	N2—H2	0.8600
C3D—H3D2	0.9600	C3'—C3A	1.513 (8)
C3D—H3D3	0.9600	C01—C07	1.499 (9)
O3—C3'	1.185 (7)	C3A—H3A	1.01 (7)
O2—C2'	1.214 (6)	C07—H07A	0.9700
N3—C2'	1.343 (7)	C07—H07B	0.9700
N3—C3A	1.452 (8)		
			
C03—C02—C01	120.1 (11)	C0'—N1—C1A	120.3 (4)
C03—C02—H02	119.9	C0'—N1—H1	119.9
C01—C02—H02	119.9	C1A—N1—H1	119.9
C3C—C3B—C3A	114.2 (7)	O2—C2'—N3	123.0 (5)
C3C—C3B—H3B1	108.7	O2—C2'—C2A	122.2 (5)
C3A—C3B—H3B1	108.7	N3—C2'—C2A	114.8 (4)
C3C—C3B—H3B2	108.7	C0'—O08—C07	118.6 (5)
C3A—C3B—H3B2	108.7	O1—C1'—N2	121.8 (5)
H3B1—C3B—H3B2	107.6	O1—C1'—C1A	121.0 (4)
C01—C06—C05	118.7 (14)	N2—C1'—C1A	117.1 (5)
C01—C06—H06	120.6	C3'—O4—H4	109.5
C05—C06—H06	120.6	N1—C1A—C1'	113.8 (4)
C3B—C3C—C3D	117.8 (13)	N1—C1A—H1A1	108.8
C3B—C3C—H3C1	107.8	C1'—C1A—H1A1	108.8
C3D—C3C—H3C1	107.8	N1—C1A—H1A2	108.8
C3B—C3C—H3C2	107.8	C1'—C1A—H1A2	108.8
C3D—C3C—H3C2	107.8	H1A1—C1A—H1A2	107.7
H3C1—C3C—H3C2	107.2	C1'—N2—C2A	123.5 (4)
C05—C04—C03	120.0 (17)	C1'—N2—H2	118.2
C05—C04—H04	120.0	C2A—N2—H2	118.2
C03—C04—H04	120.0	O3—C3'—O4	125.0 (5)
C04—C05—C06	120.7 (17)	O3—C3'—C3A	124.5 (5)
C04—C05—H05	119.6	O4—C3'—C3A	110.3 (5)
C06—C05—H05	119.6	C02—C01—C06	117.2 (8)
C04—C03—C02	123.2 (16)	C02—C01—C07	121.8 (8)
C04—C03—H03	118.4	C06—C01—C07	121.0 (9)
C02—C03—H03	118.4	O0—C0'—N1	124.4 (5)
C3C—C3D—H3D1	109.5	O0—C0'—O08	123.4 (5)
C3C—C3D—H3D2	109.5	N1—C0'—O08	112.2 (5)
H3D1—C3D—H3D2	109.5	N3—C3A—C3'	109.7 (5)
C3C—C3D—H3D3	109.5	N3—C3A—C3B	112.2 (6)
H3D1—C3D—H3D3	109.5	C3'—C3A—C3B	111.3 (6)
H3D2—C3D—H3D3	109.5	N3—C3A—H3A	107 (4)
C2'—N3—C3A	120.6 (4)	C3'—C3A—H3A	114 (4)
C2'—N3—H3	115 (4)	C3B—C3A—H3A	102 (4)
C3A—N3—H3	124 (4)	O08—C07—C01	108.0 (5)
N2—C2A—C2'	112.0 (4)	O08—C07—H07A	110.1
N2—C2A—H2A1	109.2	C01—C07—H07A	110.1
C2'—C2A—H2A1	109.2	O08—C07—H07B	110.1
N2—C2A—H2A2	109.2	C01—C07—H07B	110.1
C2'—C2A—H2A2	109.2	H07A—C07—H07B	108.4
H2A1—C2A—H2A2	107.9		
			
C3A—C3B—C3C—C3D	−171.1 (13)	C05—C06—C01—C02	1.7 (16)
C03—C04—C05—C06	−3 (3)	C05—C06—C01—C07	−178.9 (11)
C01—C06—C05—C04	0 (3)	C1A—N1—C0'—O0	14.0 (9)
C05—C04—C03—C02	4 (3)	C1A—N1—C0'—O08	−167.9 (5)
C01—C02—C03—C04	−2 (2)	C07—O08—C0'—O0	−1.1 (10)
C3A—N3—C2'—O2	0.0 (9)	C07—O08—C0'—N1	−179.3 (6)
C3A—N3—C2'—C2A	−179.2 (5)	C2'—N3—C3A—C3'	−152.6 (6)
N2—C2A—C2'—O2	5.3 (7)	C2'—N3—C3A—C3B	83.1 (8)
N2—C2A—C2'—N3	−175.5 (5)	O3—C3'—C3A—N3	−18.6 (10)
C0'—N1—C1A—C1'	76.2 (7)	O4—C3'—C3A—N3	165.6 (6)
O1—C1'—C1A—N1	17.7 (7)	O3—C3'—C3A—C3B	106.2 (8)
N2—C1'—C1A—N1	−166.6 (4)	O4—C3'—C3A—C3B	−69.7 (8)
O1—C1'—N2—C2A	0.5 (8)	C3C—C3B—C3A—N3	57.1 (9)
C1A—C1'—N2—C2A	−175.2 (4)	C3C—C3B—C3A—C3'	−66.3 (9)
C2'—C2A—N2—C1'	133.1 (5)	C0'—O08—C07—C01	−173.7 (6)
C03—C02—C01—C06	−1.0 (14)	C02—C01—C07—O08	87.8 (9)
C03—C02—C01—C07	179.6 (9)	C06—C01—C07—O08	−91.5 (9)

### Hydrogen-bond geometry (Å, º)

**Table:**

*D*—H···*A*	*D*—H	H···*A*	*D*···*A*	*D*—H···*A*
N1—H1···O3^i^	0.86	2.47	3.061 (6)	127
N2—H2···O0^ii^	0.86	2.06	2.891 (6)	164
N3—H3···O2^iii^	0.96 (7)	2.36 (7)	3.268 (6)	159 (5)
O4—H4···O1^iv^	0.82	1.83	2.593 (5)	153

Symmetry codes: (i) −*x*+2, *y*+1/2, −*z*+1/2; (ii) *x*−1/2, −*y*+1/2, −*z*; (iii) *x*+1, *y*, *z*; (iv) −*x*+2, *y*−1/2, −*z*+1/2.
