# Supplementary material for: Crystal structure of the tripeptide N-(benzyl­oxycarbon­yl)glycylglycyl-l-norvaline
Source: Acta Crystallogr E Crystallogr Commun. 2015 Feb 28;71(Pt 3):o216–7. doi: 10.1107/S205698901500393X (PMC4350747; doi:10.1107/S205698901500393X)
Supplement: Supplementary file 3 [file e-71-0o216-Isup3.docx]

**Crystal structure of the tripeptide *N*-Cbz-Gly-Gly-L-Nva**

**Supplementary data**

**Chemical context**

Norvaline is a non-proteinogenic aminoacid with an unbranched side chain. It is an isomer of the branched chain aminoacid valine. It is postulated that norvaline has been an abundant protein component during primitive stages of cell evolution (Alvarez-Carreño *et al*., 2013). Norvaline is formed as a byproduct during isoleucine fermentation from threonine by *Serratia marcescens* (Kisumi *et al*., 1976a, 1976b). The title peptide contains a Gly-Gly segment. This structural study was undertaken as part of an endeavour to understand the conformational flexibility of consecutive glycine segments in short peptides. Due to the conformational freedom of glycine residues they are increasingly found in turns (Ramakrishnan and Srinivasan, 1990). In various polymorphic forms of Tyr-Gly-Gly-Phe-Leu, the Gly-Gly segment adopts extended conformation, type-I’ β-turn and 3_10_ helical structures (Karle *et al*., 1983; Smith *et al*., 1978; Aubry *et al*., 1989). This demonstrates the conformational flexibility of consecutive glycine sequences.

**Structural commentary**

The Gly-Gly segment of the protected tripeptide has an extended conformation with Gly(1) adopting torsion angle values *φ*_1_ = 76.2(7)° and *ψ*_1_ = -166.6(4)° and Gly(2) adopting torsion angle values *φ*_2_ = 133.1(5)° and *ψ*_2_ = -175.5(5)°. The norvaline residue adopts extended conformation with torsion angle values *φ*_3_ = -152.6(6)° and *ψ*_3_ = 165.6(6)°. There are no intramolecular hydrogen bonds which stabilize the backbone conformation of the peptide molecule. The side chain of norvaline adopts *g*^+^*t* conformation.

**Supramolecular features**

The packing in the crystal structure is stabilized by four intermolecular hydrogen bonds. Molecules translated along the crystallographic a-axis associate through a hydrogen bond. The remaining three hydrogen bonds are between molecules related by 2_1_ screw symmetry. Apart from these hydrogen bonds, intermolecular aromatic π-π interactions between the phenyl rings belonging to the N-terminal protecting groups of the peptide molecules also stabilize packing in the crystal structure.

**Synthesis and crystallization**

The title compound was purchased commercially. Needle-shaped crystals of the title compound were obtained by slow evaporation from methanol/water solution.

**Refinement**

The H-atoms bonded to N3 and C3A could be located from difference Fourier maps. The remaining H-atoms were fixed geometrically in calculated positions and included in the refinement using a riding model approximation. The C-H distances were fixed at 0.97Å, 0.96Å and 0.93Å in case of hydrogens attached to methylene, methyl and aromatic carbon atoms respectively. N-H and O-H distances were fixed at 0.86Å and 0.82Å respectively. The isotropic displacement parameter U_iso_ for hydrogen atoms were set at 1.5 times the U_eq_ of the carrier atoms in case of methyl groups and hydroxyl groups. In case of hydrogens attached to aromatic ring carbons, methylene carbons and nitrogens, U_iso_ was set at 1.2 times the U_eq_ of the carrier atoms. The anisotropic displacement parameters of the carbon atoms C3A, C3B, C3C and C3D were restrained to be equal within a standard uncertainty of 0.01Å^2^ using the DELU command in *SHELXL97* (Sheldrick, 2008). In the absence of significant anomalous scattering effects, Friedel pairs were merged. The absolute configuration was known for the purchased material.


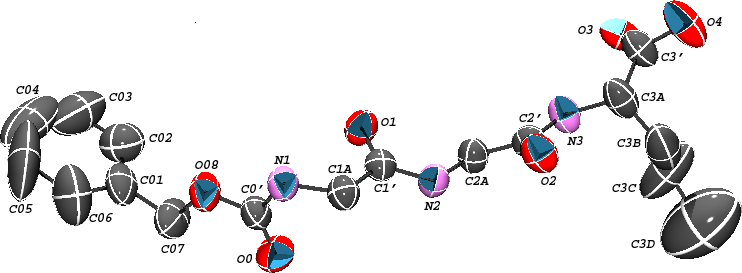


**Figure 1**: Thermal Ellipsoid plot of **NCbz-Gly-Gly-Nva-OH** drawn at 50% probability level. Hydrogen atoms are omitted for clarity.

**
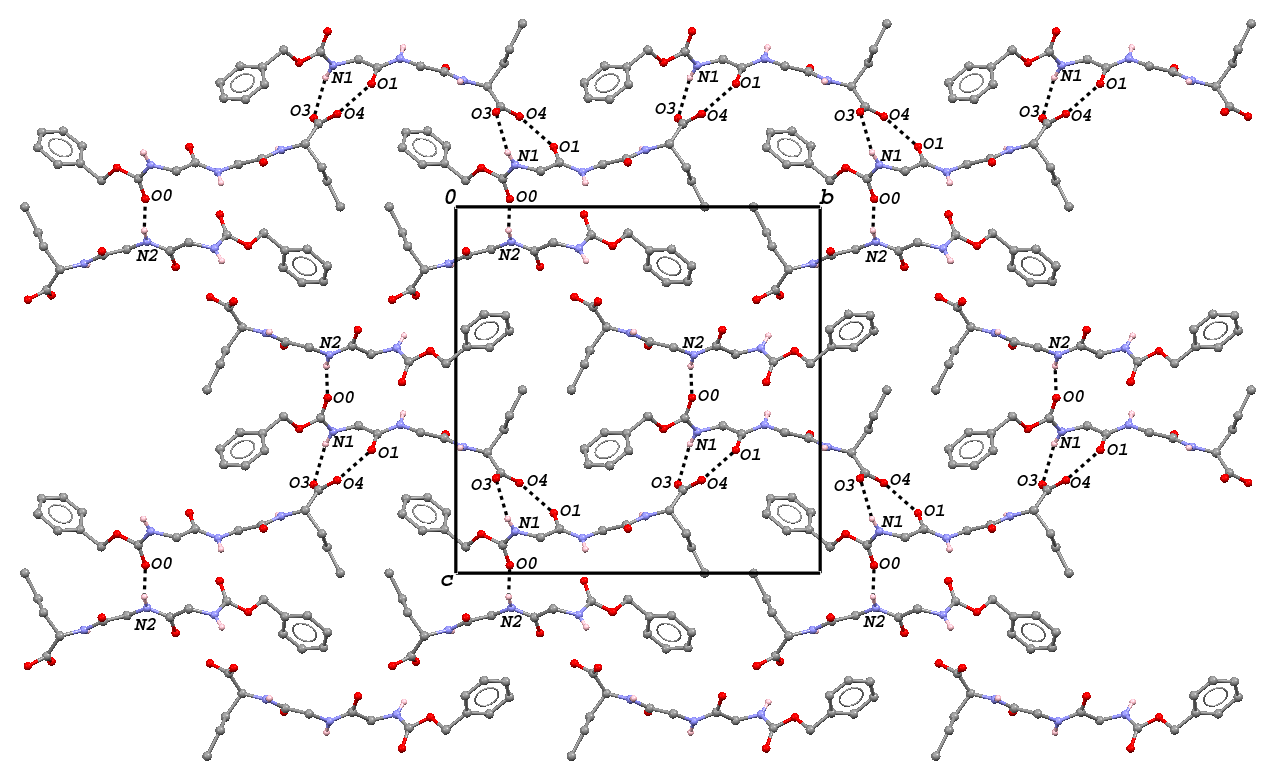
**

**Figure 2**: Packing in the crystal structure of **NCbz-Gly-Gly-Nva-OH** as viewed down the a-axis. Hydrogen bonds are represented as dotted lines. Hydrogen atoms except those involved in hydrogen bonds are omitted for clarity.


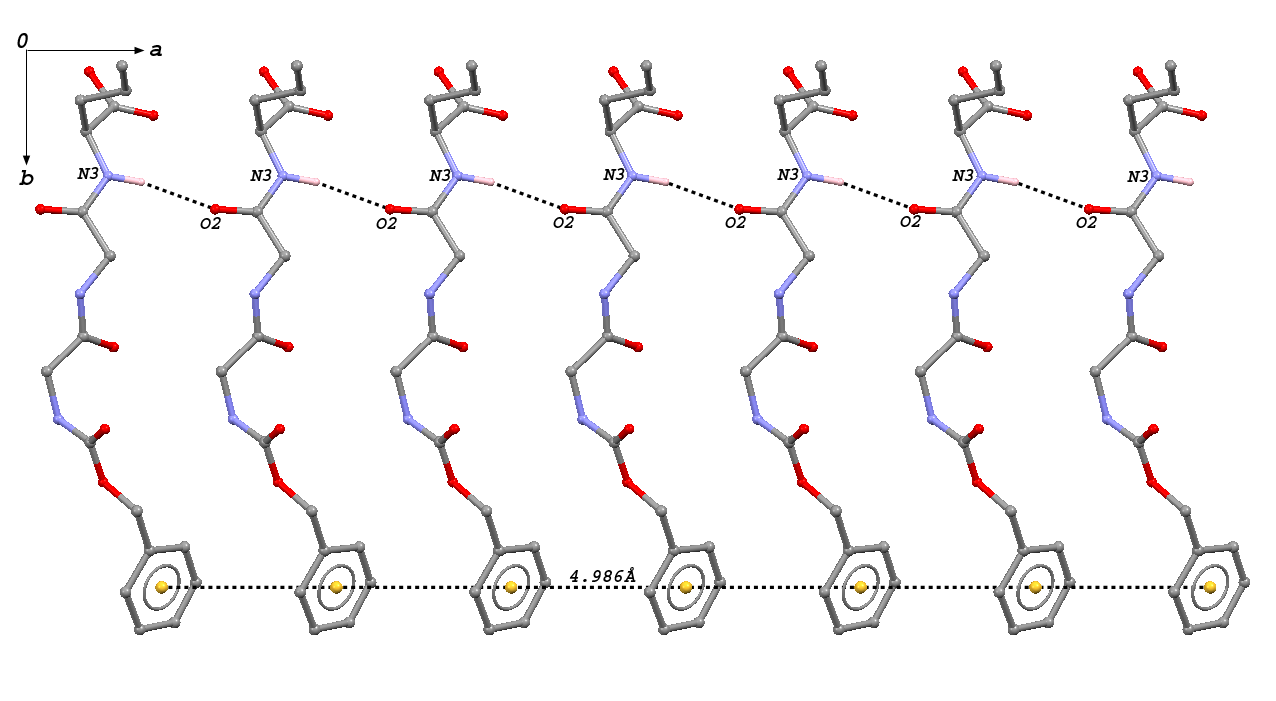


**Figure 3**: Packing in the crystal structure of **NCbz-Gly-Gly-Nva-OH** as viewed down the crystallographic c-axis. Aromatic π-π interactions and hydrogen bonds between molecules related by translation along the crystallographic a-axis are depicted. Hydrogen atoms except those involved in hydrogen bonds are omitted for the sake of clarity.
