# Supplementary figures and images for: Crystal structure of the tripeptide N-(benzyl­oxycarbon­yl)glycylglycyl-l-norvaline
Source: Acta Crystallogr E Crystallogr Commun. 2015 Feb 28;71(Pt 3):o216–7. doi: 10.1107/S205698901500393X (PMC4350747; doi:10.1107/S205698901500393X)

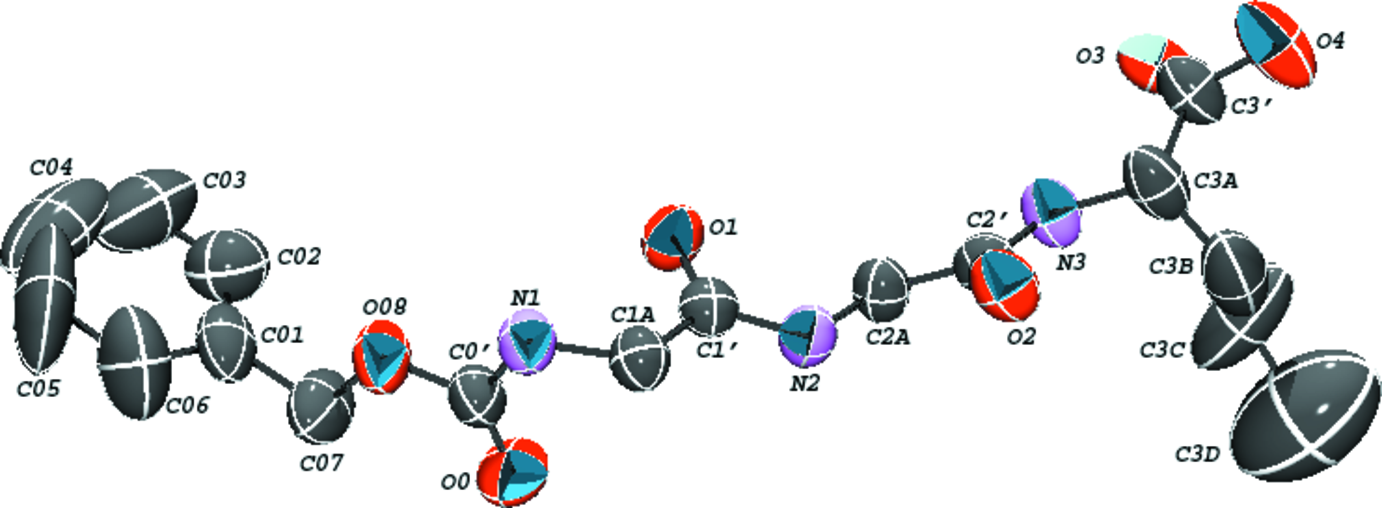

Supplement: Supplementary file 5 [file e-71-0o216-fig1.tif]

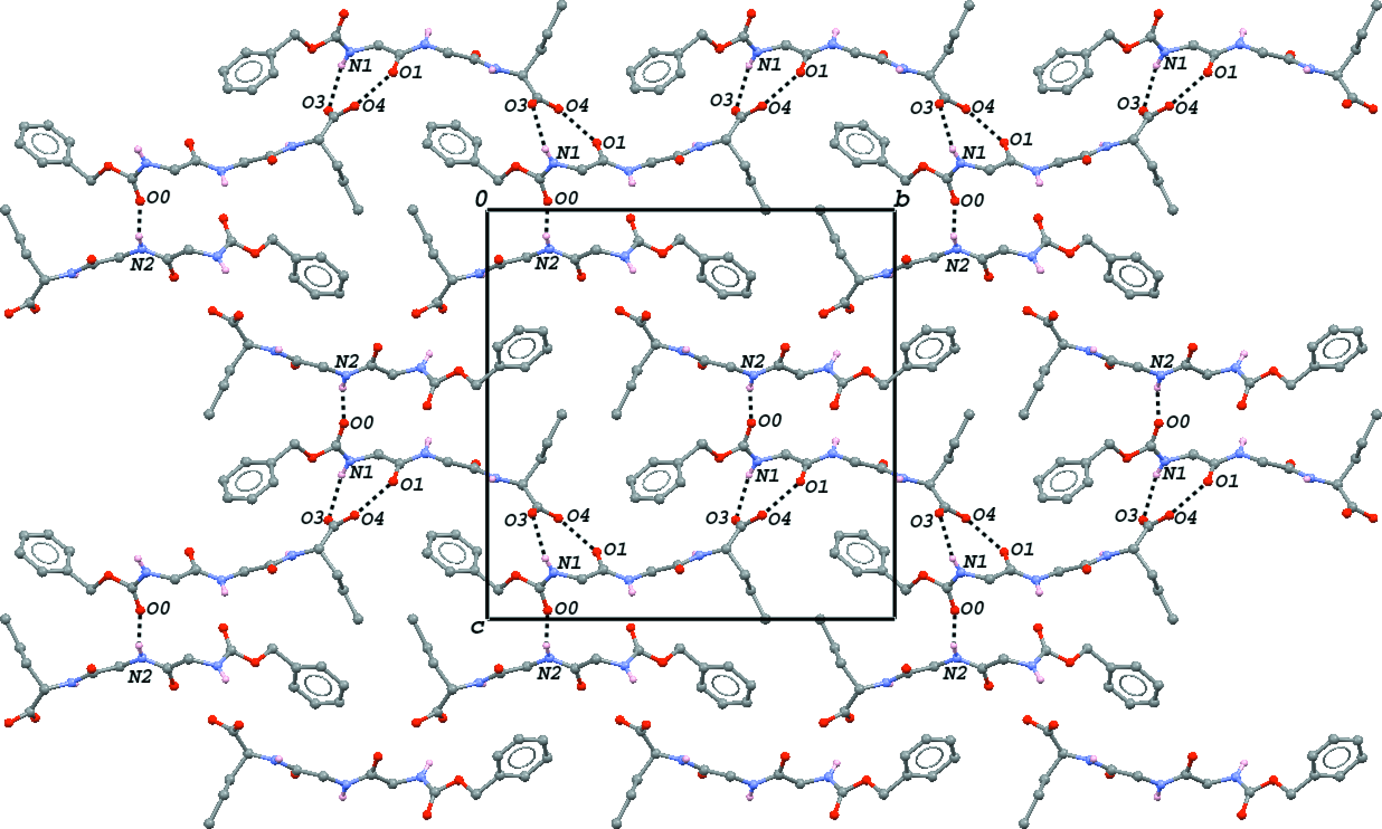

Supplement: Supplementary file 6 [file e-71-0o216-fig2.tif]
